# MAS NMR detection of hydrogen bonds for protein secondary structure characterization

**DOI:** 10.1007/s10858-020-00307-z

**Published:** 2020-03-17

**Authors:** Daniel Friedrich, Jacqueline Perodeau, Andrew J. Nieuwkoop, Hartmut Oschkinat

**Affiliations:** 1 Leibniz-Forschungsinstitut für Molekulare Pharmakologie (FMP), Robert-Rössle-Strasse 10, 13125 Berlin, Germany; 2 Institut für Chemie und Biochemie, Freie Universität Berlin, Takustrasse 3, 14195 Berlin, Germany; 3 Department of Chemistry and Chemical Biology, Rutgers University, 123 Bevier Rd., Piscataway, NJ 08854 United States; 4 Present Address: Department of Molecular and Cellular Biology, Harvard University, 52 Oxford Street, Cambridge, MA 02138 USA; 5 Present Address: Department of Cancer Biology, Dana-Farber Cancer Institute, 360 Longwood Avenue, Boston, MA 02215 USA

**Keywords:** Fast MAS, Proton detection, Hydrogen bonds, Secondary structure, Cross polarization

## Abstract

Hydrogen bonds are essential for protein structure and function, making experimental access to long-range interactions between amide protons and heteroatoms invaluable. Here we show that measuring distance restraints involving backbone hydrogen atoms and carbonyl- or α-carbons enables the identification of secondary structure elements based on hydrogen bonds, provides long-range contacts and validates spectral assignments. To this end, we apply specifically tailored, proton-detected 3D (H)NCOH and (H)NCAH experiments under fast magic angle spinning (MAS) conditions to microcrystalline samples of SH3 and GB1. We observe through-space, semi-quantitative correlations between protein backbone carbon atoms and multiple amide protons, enabling us to determine hydrogen bonding patterns and thus to identify β-sheet topologies and α-helices in proteins. Our approach shows the value of fast MAS and suggests new routes in probing both secondary structure and the role of functionally-relevant protons in all targets of solid-state MAS NMR.

**Electronic supplementary material:**

The online version of this article (10.1007/s10858-020-00307-z) contains supplementary material, which is available to authorized users.

## Introduction

One of the primary driving forces behind protein folding is the formation of hydrogen bonds characteristic for secondary structure (Dobson 2003; Jeffrey 1997; Pace et al. 2014). The ubiquitous presence of β-sheets and α-helices in proteins is a direct result of their tendency to maximize the number of hydrogen bonds. In addition, hydrogen bonds are essential in coordinating functionally-relevant water molecules and modulating interactions between biomolecules such as proteins, nucleic acids, lipids, and small molecule cofactors (Baker and Hubbard 1984; Gonen et al. 2005; Janin et al. 2008; Jones and Thornton 1996; Poornima and Dean 1995; Steitz 1990). Considering the methodological repertoire in structural biology, NMR spectroscopy is particularly well-suited to detect hydrogen atoms and their interactions with heteroatoms such as nitrogen and carbon at atomic resolution (Andreas et al. 2015; Hong et al. 2012; Zhou et al. 2012).

Magic angle spinning (MAS) solid-state NMR has become a reliable technique for structural investigations on challenging systems, such as polydisperse oligomers, fibrils, functional aggregates, and membrane proteins (Castellani et al. 2002; Loquet et al. 2013; Lu et al. 2013; Retel et al. 2017; Shahid et al. 2012; Tuttle et al. 2016; Wasmer et al. 2008). The enhancement in sensitivity gained from proton-detection under fast MAS enables the exploitation of a larger chemical shift space, thus increasing the spectral resolution and facilitating assignment of even more complex spectra while requiring smaller amounts of material. Crucial to the success of proton-detection has been the combination of high MAS frequencies (> 40 kHz) with protein deuteration while some work on fully protonated samples has been promising, especially at the highest MAS frequencies routinely available (Agarwal et al. 2014; Andreas et al. 2016; Lewandowski et al. 2011; Nieuwkoop et al. 2015; Reif 2012; Stöppler et al. 2018; Struppe et al. 2017). For larger systems, deuteration followed by back-exchange at the labile sites is extremely effective regarding sharpening proton linewidths through limiting the number of protons in the core of the protein (Akbey et al. 2010; Chevelkov et al. 2006). If protons involved in hydrogen bonds may be back-exchanged likewise, the situation seems ideal for determining which of these bonds are present in protein structure. This minimal bath of protons has additional benefits useful for acquiring structural restraints, in particular between amide protons and backbone carbons by utilizing cross polarization (CP), which we have designed an experiment to take advantage of. An added reason to be optimistic about the prospects of detecting long-range contacts between these nuclei in proton-detected experiments is the continued success of ^1^H–^13^C CP in back-exchanged perdeuterated samples. CP has long been used in solid-state NMR to gain sensitivity by utilizing the higher initial polarization and shorter relaxation times of ^1^H relative to ^13^C or ^15^N (Pines 1972). While transfers to directly bonded nuclei are most efficient, CP is a dipolar process, and thus also useful for detecting through-space interactions in the sparsely protonated context of a back-exchanged sample. Accordingly, it has been shown earlier that protons in hydrogen bonds and hydroxyl protons can be detected by solid-state MAS NMR using CP (Agarwal et al. 2010, 2013; Friedrich et al. 2020).

In the 3D (H)NCOH and (H)NCAH spectra recorded here on microcrystalline preparations of the α-spectrin SH3 domain and GB1 (for CP-based 2D ^15^N–^1^H spectra see Supplementary Figure 1), long-range contacts between ^13^CO or ^13^Cα and ^1^H^N^ are observed. We employ the resulting characteristic cross peak patterns reflecting hydrogen bonds expected for β-sheets and α-helices to analyze secondary protein structure. In addition, the ^13^CO of prolines is correlated to ^1^H^N^ resonances of residues close in space.

## Results

### Correlating multiple amide protons to carbonyls and vice versa by 2D (H)COH

**Fig. 1:**
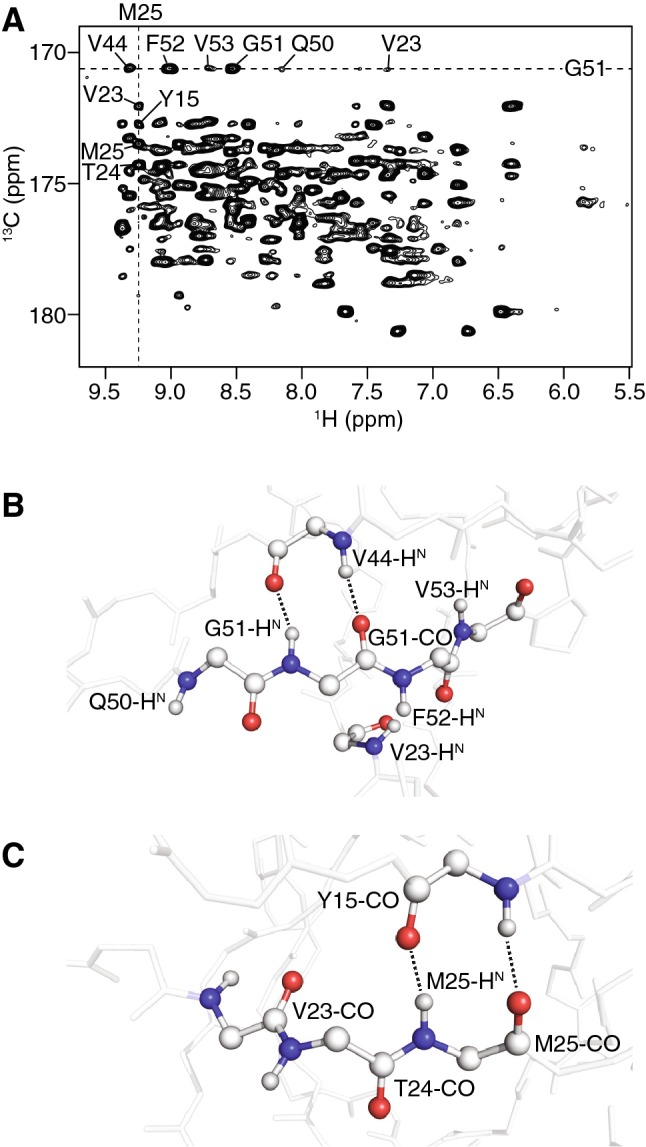
Correlations between amide protons and carbonyl carbons in SH3. **a** Solid-state NMR (H)COH 2D spectrum recorded at 40 kHz MAS with long contact times for both CP-transfer steps. Dashed lines indicate cross peaks of G51-CO and M25-H^N^. **b** Structural view of amide protons correlating to the carbonyl of G51 in the (H)CH 2D spectrum. Dashed lines indicate hydrogen bonds. **c** Carbonyls of residues close to the amide proton of M25, hydrogen bonds are indicated by dashed lines

To demonstrate the idea of CP-based detection of long-range contacts between multiple amide protons and carbonyls, we acquired a proton-detected 2D (H)COH experiment (Supplementary Figure 2) at fast MAS on a ^2^H, ^13^C, ^15^N-labeled microcrystalline sample of the SH3 domain, back-exchanged in 70% ^1^H_2_O/30% ^2^H_2_O (Fig. 1a). This experiment consists of two ^1^H–^13^C CP transfers, both of which were set to a longer contact time (4 ms) than is traditionally used. Each carbonyl is then correlated to multiple protons. A typical example is given by cross peaks involving the carbonyl resonance of G51. At the respective frequency, strong cross peaks due to amide protons of sequential residues are found (F52, V53, Q50, Fig. 1a and b). Especially where long-range cross peaks are detected, spin diffusion may add to signal intensities, which might be the case for V53 and Q50, for example (Fig. 1a and b). However, we cannot differentiate the spin diffusion and CP contributions based on the presented experiment. A hydrogen bond is indicated by the signal involving V44-H^N^ and the CO of G51, exhibiting a lower intensity than the sequential correlations due to the slightly longer distance involved. Of course, other long-range contacts also lead to cross peaks (V23). As an example for an analysis along the ^13^C dimension, the M25-H^N^ shows correlations to carbonyl signals of sequential residues (M25, T24 and V23), but also to Y15-CO which is involved in a hydrogen bond with M25-H^N^ (Fig. 1a and c). In summary, observed at the chemical shift of the carbonyl carbon, a clear pattern is obtained, with the sequential correlations involving H^N^_i±1_ being strong, and the cross-strand correlations in β-sheets indicating hydrogen bonds being not much weaker. Furthermore, this spectrum shows the promise of using long CP mixing times in deuterated samples to detect protons engaged in hydrogen bonding. The distributed magnetization still yields a selective pattern. Correlating each carbon to many protons, however, results in crowded 2D spectra, therefore we set out to design a 3D experiment which will be useful in studies of larger systems.

### Pulse sequence design of 3D (H)NCOH and (H)NCAH experiments

**Fig. 2:**
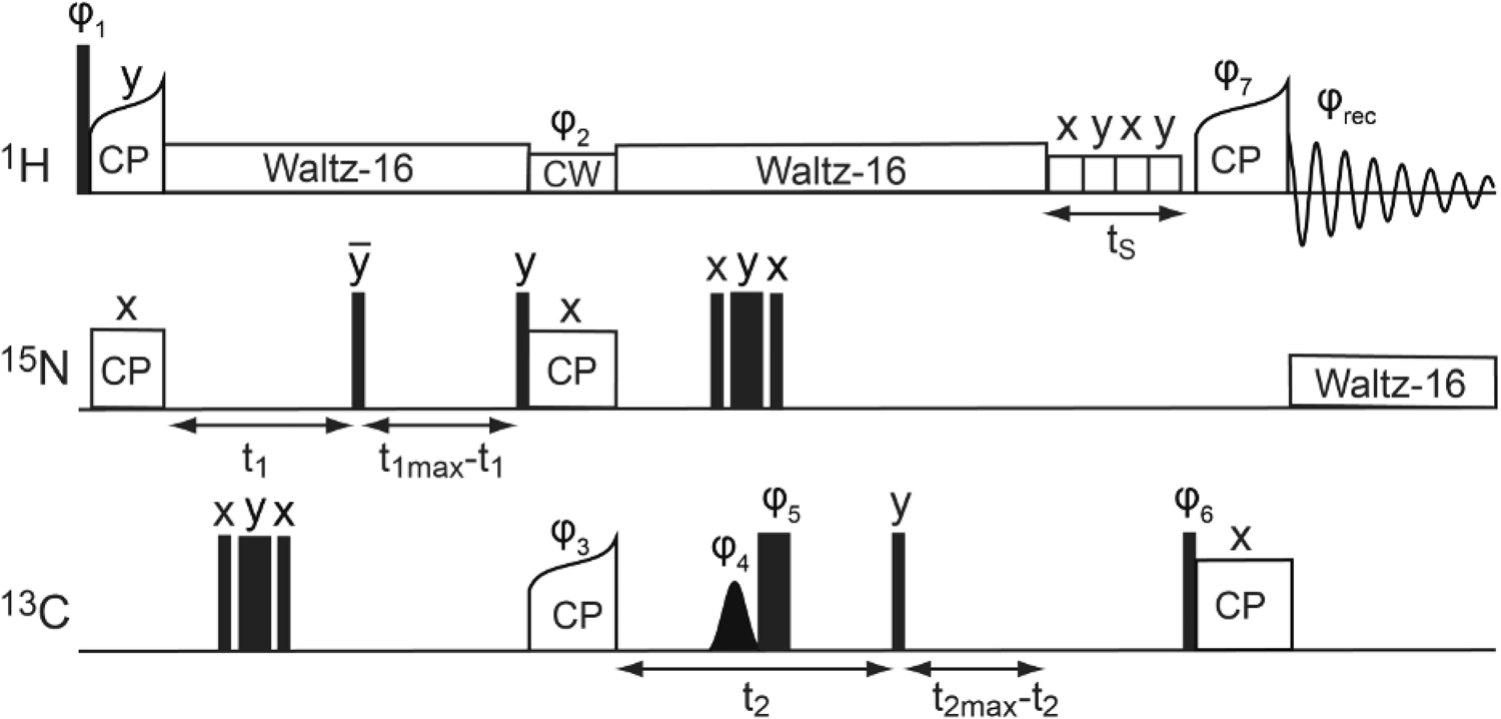
Pulse sequence for detecting hydrogen bonds. A ^15^N dimension is introduced to gain spectral resolution. To probe CO–H^N^ correlations, a long contact time is applied in the last CP step from ^13^CO to ^1^H. Both the acquisition of (H)NCOH and HN(CO)H can be used to investigate hydrogen bonds between carbonyls and amide protons. The experiment can be easily modified to collect a (H)NCAH spectrum. Constant decoupling duty cycles were used during the t_1_ and t_2_ time periods. Black, wide rectangles represent π pulses, black, narrow rectangles π/2 pulses, and the black, gaussian shape a selective π pulse. *CP* cross polarization, *CW* continuous wave decoupling, and *τ*_*S*_ MISSISSIPPI solvent suppression. The phase cycle was as follows: ϕ_1_ = 0022, ϕ_2_ = 0022, ϕ_3_ = 00002222, ϕ_4_ = 1122, ϕ_5_ = 11112222, ϕ_6_ = 13, ϕ_7_ = 1122, and ϕ_rec_ = 13201320

A ^15^N-dimension can be used to resolve long-range correlations observed in crowded (H)CH 2D spectra employing long CP contact times. The corresponding 3D pulse sequence, which has the same form as the suite of Hα-detected assignment spectra recently introduced by the Pintacuda group (Stanek et al. 2016), can be used for detecting through-space contacts between protons and both Cα and CO nuclei. Using the pulse sequence shown in Fig. 2, data from ^2^H, ^15^N, ^13^C-labeled samples were recorded at 40 kHz MAS for SH3 and 37 kHz MAS for GB1, but the experiment may be used at MAS frequencies up to 110 kHz and above.

The essential unit of the proposed experiment is a ^13^CO–^1^H CP step of about 4–6 ms, to ensure detection of distances exceeding 3 Å. The most straightforward implementation of an appropriate 3D experiment would be to evolve the first ^1^H dimension to achieve an HCH 3D. This approach would have limitations however, as the chemical shift dispersion of both ^1^H and ^13^CO are not particularly large. If, however, we introduce a ^15^N dimension, we could gain additional spectral resolution. By exchanging the ^13^C and ^15^N dimensions of the traditional (H)CONH pulse sequence, hydrogen-bonded protons can be observed in the direct dimension via an (H)NCOH 3D (Fig. 2). Depending on the resolution achieved in the indirect dimensions, evolution of the ^15^N dimension (instead of ^13^CO) resulting in an HN(CO)H experiment could prove useful.

To observe correlations involving the ^13^Cα resonances, this experiment can be modified by utilizing a selective CP transfer from nitrogen to ^13^Cα followed by evolution of the ^13^Cα frequencies. This version should be acquired as an (H)NCAH 3D to make benefit of the dispersion of the ^13^Cα resonances. Similar to the experiment involving ^13^CO, it is important to apply a long CP contact time (about 4–6 ms) for the transfer from carbon to protons before acquisition to probe long-range contacts of the frequency-labeled N-Cα pair to protons nearby.

We tested both the (H)NCOH and the (H)NCAH experiments on microcrystalline samples of SH3 and GB1 to evaluate observed signals towards their long-range correlations.

### Expected hydrogen bonding and signal patterns in secondary structure elements of proteins

**Fig. 3:**
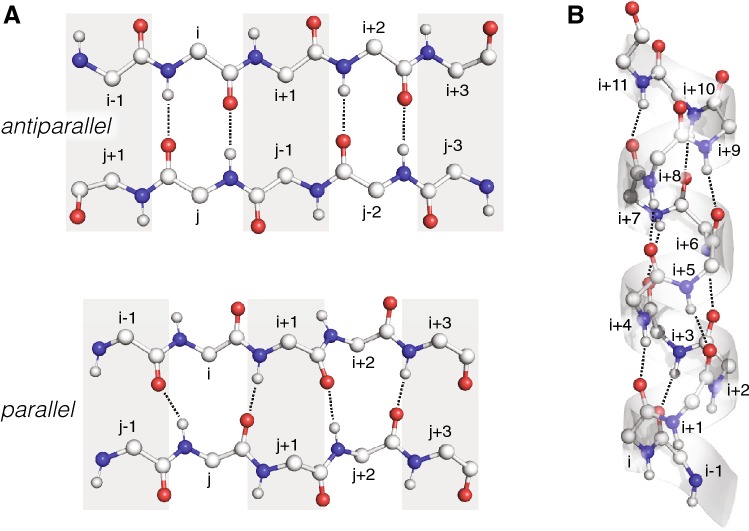
Characteristic hydrogen bond patterns in secondary structure elements. Nitrogen atoms are shown as blue, oxygen as red, hydrogen atoms as small white, and carbon atoms as big white spheres. **a** Different hydrogen bonding patterns are established in idealized antiparallel β-sheets (between every other CO_i_ and H^N^_j_ and vice versa, top panel) and parallel β-sheets (between every other CO_i−1_ and H^N^_j_, and CO_j_ and H^N^_i+1_, bottom panel) [PDB codes 2LNQ and 2LBU, respectively (Qiang et al. 2012; Schütz et al. 2011)]. **b** In an ideal α-helix [PDB code 4U1H (Kløverpris et al. 2015)], hydrogen bonds are formed between CO_i_ and H^N^_i+4_

The two pulse sequences deliver slightly different patterns. In case of the experiment correlating ^13^CO resonances, the partner directly involved in a hydrogen bond is monitored by its chemical shift, and the correlation represents a comparably short distance, albeit slightly longer than to sequential sites. In case of the (H)NCAH experiment, the hydrogen bond is not observed directly, but correlations involving the H^N^ of a given hydrogen bond are accessible, and the corresponding distances are longer. For this reason, the pattern is less selective, but might provide individual cross peaks that help distinguishing hydrogen bonding patterns. Hence, both spectra viewed together display the overall situation in a clearer way. The presence of β-sheet or α-helix as secondary structure elements leads to different closest amide protons to carbonyl- or α-carbons (Fig. 3), with characteristic distances (Supplementary Table 1) that may be reflected in cross peak intensities.

In (H)NCOH experiments, the expected cross peak of the CO to the amide proton of the same amino acid and the signals correlating CO_i_ and H^N^_i+1_ are trivial but serve for orientation. Different signal patterns are then expected in antiparallel and parallel β-sheets. In the antiparallel case, two hydrogen bonds between opposing residues i and j appear at every second pair along the two polypeptide chains (Fig. 3a, top panel), leading to an additional cross peak at the chemical shifts of CO_i_ and H^N^_j_, respectively CO_j_ and H^N^_i_. Neglecting relaxation effects, the intensities of the sequential cross peaks are expected to be larger than the cross-strand ones. Comparing 2D slices taken at the chemical shifts of CO_i_/N_i+1_ and CO_j_/N_j+1_ yields the two respective pairs of cross peaks with like amide proton frequencies (i and j). The same pattern may be observed for residues i + 2 and j − 2, and so forth, enabling the delineation of β-sheet topology. Since the other cross-strand carbonyl atoms are much further away, this is the dominant inter-strand feature for antiparallel β-sheets. In the parallel case, the hydrogen bonds involving H^N^_j_ and CO_j_ reach out to CO_i−1_ and H^N^_i+1_, respectively (Fig. 3a, bottom panel). The respective cross peaks should have again slightly lower intensities than the sequential peaks. Now, there are not two cross peaks expected with common proton frequencies associated with residues i and j, but the 2D slice taken at CO_j_/N_j+1_ shows a correlation to H^N^_i+1_, and likewise the one at CO_i−1_/N_i_ exhibits the chemical shift of H^N^_j_. Both types of β-sheets may be distinguished by different patterns in the (H)NCAH experiment, where for the parallel β-sheet a dominant inter-strand feature occurs, a cross peak involving the chemical shifts of Cα_i_/N_i_ and the amide proton chemical shift of H^N^_j_. In the contrary, all inter-strand distances in antiparallel β-sheets are much longer (4.2 Å) than the sequential ones as compared to the parallel case (3.3 Å for Cα_i_···H^N^_j_) (Supplementary Table 1). Due to spin diffusion effects, the situation might be different when non-deuterated proteins are used.

α-Helices typically contain hydrogen bonds between CO_i_ and H^N^_i+4_ (Fig. 3b). However, seen from the CO, quite a number of sequential amide protons are in a distance range of 3.2 Å (H^N^_i_, H^N^_i+2_, H^N^_i+3_, and H^N^_i+4_), except for the trivial one (CO_i_···H^N^_i+1_ = 2.0 Å) (Supplementary Table 1). This is expected to result in a larger number of similarly intense cross peaks in (H)NCOH spectra involving all sequential residues around the H^N^ and CO sites, from residues i to i + 4. Since amide protons in α-helices show a smaller chemical shift dispersion, the massive appearance of cross peaks may lead to a strong superposition of signals. On the other hand, distances between α-carbons and amide protons vary a little more, and the correlation involving Cα_i_ and H^N^_i+3_ may be a slightly stronger feature due to the distance of 3.8 Å as compared to 4.2 Å for Cα_i_ and H^N^_i+4_ (Supplementary Table 1).

### Detection of hydrogen bonds and long-range contacts in SH3

**Fig. 4:**
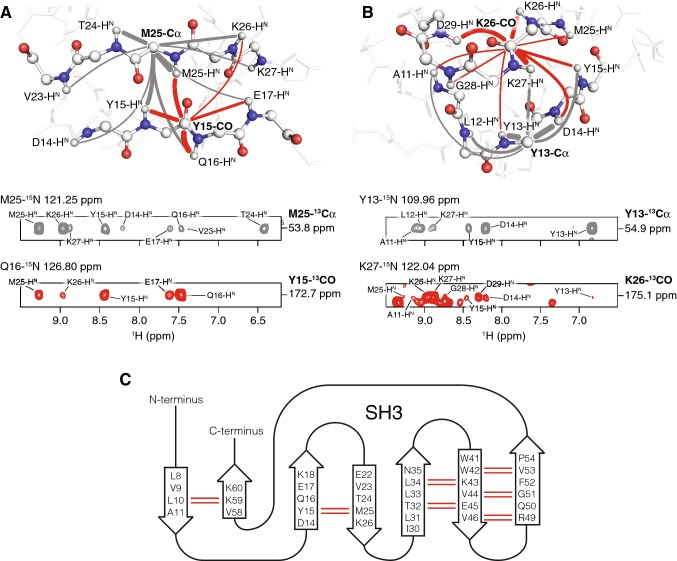
Hydrogen bond and long-range contact patterns detected in secondary structure elements of SH3. **a** β-sheet and **b** turn-like structure in microcrystalline SH3. (H)NCAH and (H)NCOH correlations are shown in grey and red, respectively. Nitrogen atoms are shown as blue, oxygen as red, hydrogen atoms as small white and carbon atoms as big white spheres. The lines in the structural illustration indicate observed interactions and their thickness reflects the corresponding signal intensities. **c** Secondary structure of SH3 [based on PDB code 1U06 (Chevelkov et al. 2005)] and β-sheet backbone hydrogen bonds detected with the (H)NCOH experiment indicated by red lines

A characteristic example for correlations yielding such constraints for secondary structure analysis is given by the cross peaks involving the residues Y15 and M25 in SH3 (Fig. 4a). In the (H)NCAH experiment, correlations of the N-Cα pair of M25 to multiple proton sites can be detected (grey spectrum and grey lines). As CP is based on dipolar coupling that depends on distance and dynamics, the cross peak intensities can be used to semi-quantitatively estimate the distances between the atomic nuclei involved as indicated by the thickness of the correlations drawn in the figures. The strongest signals result from interactions of the M25-Cα with the amide protons of M25 and the neighboring residue T24. Structurally more relevant are correlations with signals of amino acids in the next β-strand involving the amide protons of D14, Y15, Q16 and E17. The intensity of these cross peaks is much lower than the M25 and T24 signals due to the longer distances involved. In the (H)NCOH spectrum, signals reflecting amide protons near the Y15-CO can be observed (red spectrum and red lines). These include structurally trivial correlations with the amide proton signal of Y15 and the sequentially connected residues Q16 and E17. The cross peak to the amide proton of M25 reflects a hydrogen bond between the amide group of M25 and the Y15-carbonyl. As indicated in Fig. 4a, such cross peaks exhibit strong intensities. This shows the great value of the proposed experiment to detect hydrogen bonds in proteins. In addition, a weak long-range correlation to the amide proton of K26 is observed.

The situation is different in a turn-like structure in SH3 (Fig. 4b). Here, contacts between Y13-Cα and the amide protons of A11, L12, Y13, D14 and Y15 can be observed (grey spectrum and grey lines). K27-H^N^ is the only amide proton of a residue that is not close in sequence but still yielding a cross peak to Y13-Cα. The carbonyl of K26, however, shows multiple long-range interactions to amide protons of A11, Y13, D14, Y15, M25, K26, K27, G28 and D29 which are close in space (red spectrum and red lines). In contrast to the β-sheet, no hydrogen bonds exist between amide groups and carbonyls due to the less defined secondary structure in this protein region and thus a hydrogen bond-reflecting cross peak is not observed. This shows how the proposed experiment can be used to identify secondary structure elements.

In the SH3 domain, three antiparallel β-sheets exist, consisting of seven β-strands in total (Fig. 4c). In the three-dimensional (H)NCOH spectrum, the complete hydrogen bonding pattern in these β-sheets is observed. This includes, for example, L10···K59 with the expected correlations between L10-CO and K59-H^N^, and L10-H^N^ and K59-CO (Supplementary Figure 3). Such a cross peak pattern corresponds to the hydrogen bonds formed in an antiparallel β-sheet (Fig. 3a, top panel). The same is observed for the second β-sheet, for which signals reflecting the two hydrogen bonds between Y15 and M25 are detected (Fig. 4a and Supplementary Figure 3). This β-sheet is slightly twisted, therefore the CO and H^N^ of E17 and V23 do not form a hydrogen bond and thus signals cannot be observed. The third β-sheet that is formed by three β-strands shows hydrogen bonding between the CO and H^N^ atoms of ten amino acids: hydrogen bonds of four residues are found between the first two β-strands (T32···E45 and L34···K43), and of six between the second and third β-strands (W42···V53, V44···G51 and V46···R49) (Supplementary Figure 3). In this third β-sheet, the characteristic hydrogen bonding pattern for the antiparallel case is again observed.

Another value of the long CP contact time employed in the experiment presented here is the possibility to correlate the CO of a proline to amide protons close in space as shown for P54 in SH3 (Supplementary Figure 4). Such correlations close in sequence or in space facilitate the assignment of proline carbonyls and allows detection of their hydrogen bonds.

### Analysis of the α-helical hydrogen bonding pattern in GB1

**Fig. 5:**
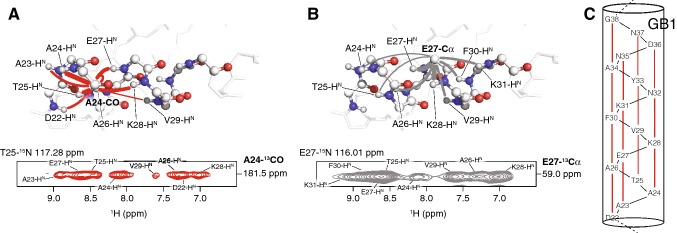
α-Helical hydrogen bonds and long-range contacts detected in GB1. Nitrogen atoms are shown as blue, oxygen as red, hydrogen atoms as small white and carbon atoms as big white spheres. The lines in the structural illustration indicate observed interactions and their thickness reflects the corresponding signal intensities. **a** The (H)NCOH experiment yields correlations between carbonyls and amide protons in close proximity (red spectrum and red lines). **b** Long-range contacts between Cα and amide protons can be observed with the (H)NCAH experiment shown in grey. **c** Hydrogen bonds (red lines) detected with the (H)NCOH experiment in the α-helix of GB1 (PDB code 2QMT, Frericks Schmidt et al. 2007)

To test whether the presented experiment yields cross peaks reflecting hydrogen bonds in an α-helix, we used a microcrystalline sample of GB1 as SH3 does not have an α-helix (Figs. 4 and 5). GB1 is an excellent model system for solid-state MAS NMR studies and has one α-helix involving residues D22 to G38 (Zhou et al. 2007). As an example, the correlations of A24-CO to amide protons nearby are shown (Fig. 5a). These include D22-H^N^, A23-H^N^, A24-H^N^, T25-H^N^, A26-H^N^, E27-H^N^, V29-H^N^, and K28-H^N^, the latter forming a hydrogen bond to the carbonyl of A24 that is characteristic for an α-helix (CO_i_···H^N^_i+4_, see Fig. 3b). The intensity of this cross peak is the second highest of these observed correlations; as expected only the signal involving A24-CO and A24-H^N^ exhibits a somewhat higher intensity as these two nuclei are slightly closer in space than A24-CO and K28-H^N^ as measured in the crystal structure of GB1 (3.2 Å versus 3.4 Å) (PDB code 2QMT, Frericks Schmidt et al. 2007). In comparison to β-sheets, α-helices are more compact, with a higher density of interactions between ^1^H^N^, ^1^H^α^ and ^1^H^β^ spins in a non-deuterated protein, and especially between ^1^H^N^ in the deuterated case, thus showing a larger number of substantial proton homonuclear dipolar couplings. Therefore, intensities of signals associated with residues located in α-helices are more likely modulated by proton spin diffusion and relaxation effects, impairing the immediate relation of distance and cross peak intensity. However, the characteristic, repetitive occurrence of CO_i_···H^N^_i+4_ contacts is still sufficient to unambiguously differentiate between α-helices and β-sheets. In the (H)NCAH spectrum, a number of long-range contacts can be detected as seen for example at E27-Cα (Fig. 5b). It shows cross peaks to A24-H^N^, T25-H^N^, A26-H^N^, E27-H^N^, K28-H^N^, V29-H^N^, F30-H^N^, and K31-H^N^. Analogous to the data for SH3, these correlations are useful to validate assignments of resonances of the nuclei involved. Using the (H)NCOH experiment, we have detected for each carbonyl of the protein backbone a hydrogen bond to an amide proton in the α-helix of GB1 (Fig. 5c, Supplementary Figure 5). In each case, these signals reflect the characteristic hydrogen-bonding pattern between CO_i_ and H^N^_i+4_ as expected for an α-helix (Fig. 3b).

## Discussion

In summary, we present three-dimensional, proton-detected solid-state MAS NMR experiments that resolve through-space contacts of protons to carbons. As an (H)NCOH experiment, it can be used to explore hydrogen bonds for secondary structure characterization. This is especially useful to identify α-helices and to distinguish parallel and antiparallel β-sheets through the respective hydrogen bonding patterns. As we show, the expected hydrogen bonds in the α-helix and antiparallel β-sheets can be detected in GB1 and SH3, respectively. The experiment gives also access to the chemical shift of proline carbonyls, which is not the case in conventional proton-detected solid-state MAS NMR experiments. In addition to investigating hydrogen bonds, the experiment can be used to collect long-range contacts by applying the modified (H)NCAH version.

The characteristic distances between ^1^H^N^, ^1^H^α^ and ^1^H^β^ in α-helices and β-sheets lead to different spin system topologies, especially when deuterated proteins are used where ^1^H^α^ and ^1^H^β^ atoms are replaced by ^2^H and only ^1^H^N^ signals are present. In β-sheets, the shortest distances between two amide protons, each in one of the opposing strands, are 3.4 Å and 3.7 Å in the antiparallel and parallel case, respectively. All other backbone amide protons are at least 4.3 Å away from one another, and exchangeable amino acid side chain protons mostly as well. For this reason, those two amide protons may be considered as an isolated two-spin system in a very coarse-grained approximation. Effects of spin diffusion should be small. In an α-helix, on the other hand, all sequentially neighbored amide protons are distant by about 2.8 Å, yielding a row of like distances along the helix, and therefore considerable effects should be observed that can be termed spin diffusion, as apparent through correlations between sequentially quite distant amide protons.

In future studies, the experiment may also be used to investigate hydrogen bonds between amino acid side chains such as aspartate/glutamate and other residues, for example arginine side chains. Work in this direction has been done by the Reif group, who detected hydrogen bonds between side chains in SH3 (CO···HO–C), and between glutamate carboxyl groups and histidine imidazole nitrogen in the amyloid β-peptide (Agarwal et al. 2010, 2013). In the case of side chains, however, the herein presented experiment needs to be modified since it features a ^15^N-filter that does not allow to observe signals of ^13^Cγ and ^13^Cδ in aspartate and glutamate, respectively. Similarly, the ^13^CO-filter prevents magnetization transfer involving the guanidinium group of arginine and thus the observation of this moiety as hydrogen bonding partner. By adding a ^13^C–^13^C mixing step, such correlations could be collected in a (H)CCOH-type experiment. Alternatively, it could be extended to an (H)N(H)COH experiment to still make use of the ^15^N chemical shift.

Finally, we propose that the hydrogen bonding patterns obtained by our approach can be used as constraints for structure determination. Complemented by chemical shift information, they may be sufficient to derive preliminary low-resolution structures. Contacts reflecting hydrogen bonds in the protein backbone will be certainly helpful to more accurately define the overall protein topology, as has been shown for example in the case of the β-barrel membrane protein OmpG (Retel et al. 2017). In general, not only the donor–acceptor distance defines a hydrogen bond, but also the angle between the two. Our presented experiments do not allow for measuring such angles. However, as NMR structure calculation programs and NMR assignment procedures do not consider them explicitly, experimental information of hydrogen bond angles are not discussed in our present study. Of course, they may have an overall effect on the distance between donor and acceptor atom, yet the error introduced is very small.

## Materials and methods

### Sample preparation and NMR measurements of SH3

The protein was expressed and purified as described in Akbey et al. and Nieuwkoop et al. and the proton level was adjusted using a ^1^H_2_O/^2^H_2_O mixture of 70% ^1^H_2_O (Akbey et al. 2010; Nieuwkoop et al. 2015).

All experiments were recorded on a 900 MHz Avance III Bruker NMR spectrometer equipped with a 1.9 mm four-channel (HCND) MAS solid-state probe using a microcrystalline ^2^H, ^13^C, ^15^N-labeled SH3 sample (70% re-protonated at exchangeable sites). The variable temperature was set to 240 K and the MAS frequency to 40 kHz for all experiments, and π/2 pulses of 100 kHz for ^1^H, 50 kHz for ^13^C and 41.67 kHz for ^15^N were used. 140 ms of MISSISSIPPI water suppression (Zhou and Rienstra 2008) and WALTZ-16 ^1^H decoupling (Shaka et al. 1983) during indirect evolution periods was applied in all experiments.

For the (H)COH 2D experiment, a contact time of 4 ms was used for both CP steps. Linear ramps of 100–75% and 80–100% were applied on ^1^H during the first and second CP transfers, respectively. The carrier was set to 174.6 ppm on ^13^C during the experiment. Both CP steps were optimized around 60 kHz for ^1^H and 20 kHz for ^13^C to fulfill the n = 1 Hartmann-Hahn condition. 4096 data points at a sweep width of 50 kHz and a maximum acquisition time of 41 ms were acquired in the direct ^1^H dimension. 256 T_2_ increments at a sweep width of 6.67 kHz were collected in the indirect ^13^C dimension, corresponding to a maximum acquisition time of 19.2 ms. An interscan delay of 1 s was used, and 4 scans per slice were acquired.

For the (H)NCOH 3D experiment, a contact time of 1.5 ms for the ^1^H–^15^N CP transfer was applied, optimized around 60 kHz for ^1^H with a linear 100–80% ramp and 20 kHz for ^15^N. A contact time of 10 ms was used for the ^15^N–^13^CO CP, optimized around 30 kHz for ^15^N with a tangential 40–60% shape and 10 kHz for ^13^CO. The ^13^CO–^1^H CP transfer was the same as in the (H)COH 2D experiment. 4 scans with an interscan delay of 1 s were recorded. The ^13^C carrier was set to 174.6 ppm and for ^15^N to 115.7 ppm. 96 increments were collected in both the ^15^N dimension (sweep width 3.33 kHz, 14.4 ms acquisition time) and the ^13^C dimension (sweep width 5 kHz, acquisition time of 9.6 ms). 4096 data points with a sweep width of 50 kHz and 41 ms acquisition time were recorded in the direct ^1^H dimension.

The (H)NCAH experiment was acquired with the same experimental conditions, except for using specific ^15^N–^13^CA and ^13^CA–^1^H CP transfers with the ^13^C carrier set to 55.2 ppm. In this experiment, 192 slices were collected for ^13^C at a sweep width of 10 kHz, yielding a maximum acquisition time of 9.5 ms in the carbon dimension.

### Sample preparation and NMR measurements of GB1

GB1 was expressed, purified, and prepared as a microcrystalline sample as previously described (Franks et al. 2005).

All experiments were recorded on a 600 MHz Avance III Bruker NMR spectrometer equipped with a Bruker MAS 3 unit and a 1.6 mm HXYD probe (Phoenix NMR) tuned to HCND using 7.8 mg microcrystalline ^2^H, ^13^C, ^15^N-labeled GB1 (100% re-protonated at exchangeable sites). The variable temperature was set to 288 K and the MAS frequency to 37 kHz for all experiments, and π/2 pulses of 161.3 kHz for ^1^H, 125.0 kHz for ^13^C and 92.6 kHz for ^15^N were used. 200 ms of MISSISSIPPI water suppression (Zhou and Rienstra 2008) and WALTZ-16 ^1^H decoupling (Shaka et al. 1983) during indirect evolution periods was applied in all experiments.

For the (H)NCOH 3D experiment, a contact time of 3.5 ms for the ^1^H–^15^N CP transfer was applied, optimized to 94.3 kHz for ^1^H with a rectangular pulse and 58.6 kHz for ^15^N with a tangential 85–100% ramp. A contact time of 8 ms was used for the ^15^N–^13^CO CP, optimized to 60.8 kHz for ^15^N with a rectangular pulse and 98.7 kHz for ^13^CO with a 90–100% tangential shape. The ^13^CO–^1^H CP transfer was 4 ms and optimized to 64.1 kHz for ^13^C with a 90–100% tangential ramp and 93.4 kHz for ^1^H with a rectangular shape. 6 scans with an interscan delay of 1 s were recorded. The ^13^C carrier was set to 169.00 ppm and for ^15^N to 120.12 ppm. 48 increments were collected in the ^15^N dimension (sweep width 2.5 kHz, 9.6 ms acquisition time) and 128 increments were collected in the ^13^C dimension (sweep width 6.2 kHz, acquisition time of 10.4 ms). 2048 data points with a sweep width of 25 kHz and 41 ms acquisition time were recorded in the direct ^1^H dimension.

The (H)NCAH experiment was acquired with the following experimental conditions. The interscan delay was set to 1 s. The ^15^N–^13^CA CP transfer (optimized to 24.6 kHz for ^15^N with a rectangular pulse and 8.6 kHz for ^13^CA with a 90–100% tangential shape) was done with the ^13^C carrier set to 52.00 ppm and a contact time of 9 ms. In this experiment, 128 slices were collected for ^13^C at a sweep width of 9.25 kHz, yielding a maximum acquisition time of 6.9 ms in the carbon dimension. 48 points were collected in the ^15^N dimension with a sweep width of 3.1 kHz. The total acquisition time was 7.8 ms. 8 scans were recorded in the ^1^H dimension and the acquisition time was 41 ms (2048 points).

## Electronic supplementary material

Below is the link to the electronic supplementary material.

Supplementary file1 (PDF 1334 kb)
