# Supplementary material for: MAS NMR detection of hydrogen bonds for protein secondary structure characterization
Source: J Biomol NMR. 2020 Mar 17;74(4):247–56. doi: 10.1007/s10858-020-00307-z (PMC7211791; doi:10.1007/s10858-020-00307-z)
Supplement: Supplementary file 1 — Supplementary file1 (PDF 1334 kb) [file 10858_2020_307_MOESM1_ESM.pdf]

Supplementary Information

MAS NMR detection of hydrogen bonds for protein secondary structure characterization

Daniel Friedrich[1,2,§], Jacqueline Perodeau[3], Andrew J. Nieuwkoop[3,*], and Hartmut Oschkinat[1,2,*]

[1] Leibniz-Forschungsinstitut für Molekulare Pharmakologie (FMP), Robert-Rössle-Strasse 10, 13125 Berlin, Germany
[2] Freie Universität Berlin, Institut für Chemie und Biochemie, Takustrasse 3, 14195 Berlin, Germany
[3] Rutgers University, Department of Chemistry and Chemical Biology, 610 Taylor Road, Piscataway, NJ 08854, USA
[§] present address: Harvard University, Department of Molecular and Cellular Biology, 52 Oxford Street, Cambridge, MA 02138, USA, and Dana-Farber Cancer Institute, Department of Cancer Biology, 360 Longwood Avenue, Boston, MA 02215, USA

* Corresponding authors: Andrew J. Nieuwkoop (an567@chem.rutgers.edu) and Hartmut Oschkinat (oschkinat@fmp-berlin.de)

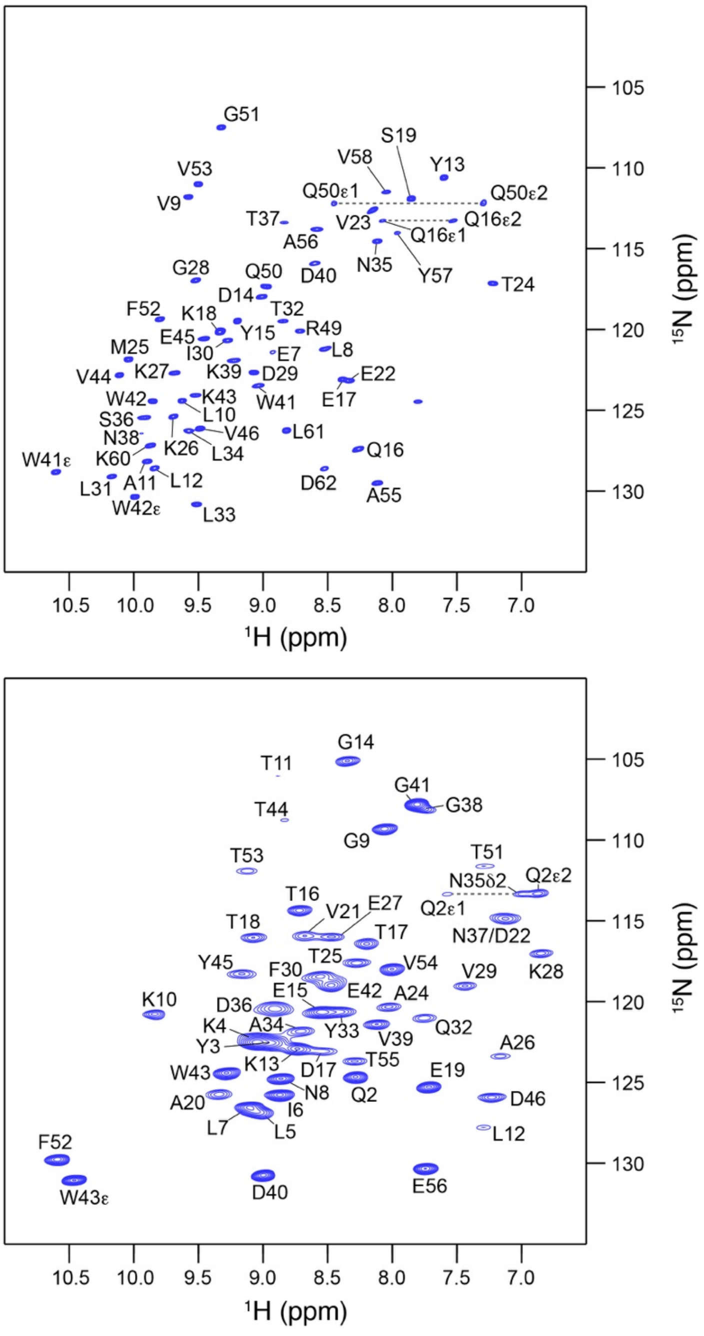

Supplementary Figure 1. Two-dimensional ^{15}N-^{1}H spectra of SH3 and GB1. To monitor sample conditions and transfer assignments, we recorded proton-detected, cross polarization-based (H)NH correlations of microcrystalline, ^{2}H,^{13}C,^{15}N-labeled SH3 (top panel, acquired at 60 kHz MAS, 70% re-protonated), and GB1 (bottom panel, acquired at 37 kHz MAS, 100% re-protonated) samples. The assignment of amide backbone signals is indicated in both cases.

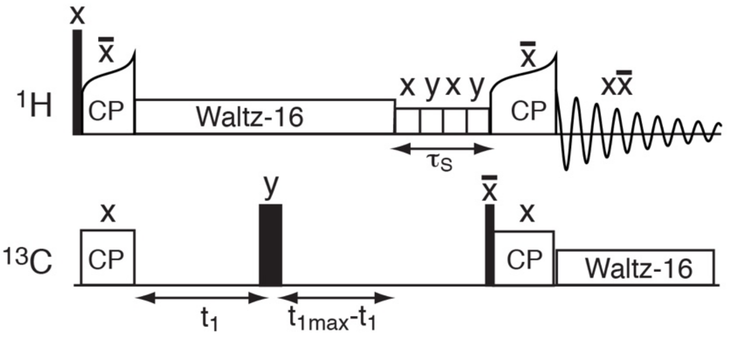

Supplementary Figure 2. Pulse sequence of the 2D (H)COH experiment. To record a 2D, proton-detected ^{13}C-^{1}H spectrum for correlating multiple amide protons with carbonyls and *vice versa*, we employed a conventional (H)COH pulse sequence with both CP transfers set to 4 ms. The black, wide rectangle represents a π pulse, and black, narrow rectangles $\pi/2$ pulses. CP = cross polarization, and τ_S = water suppression.

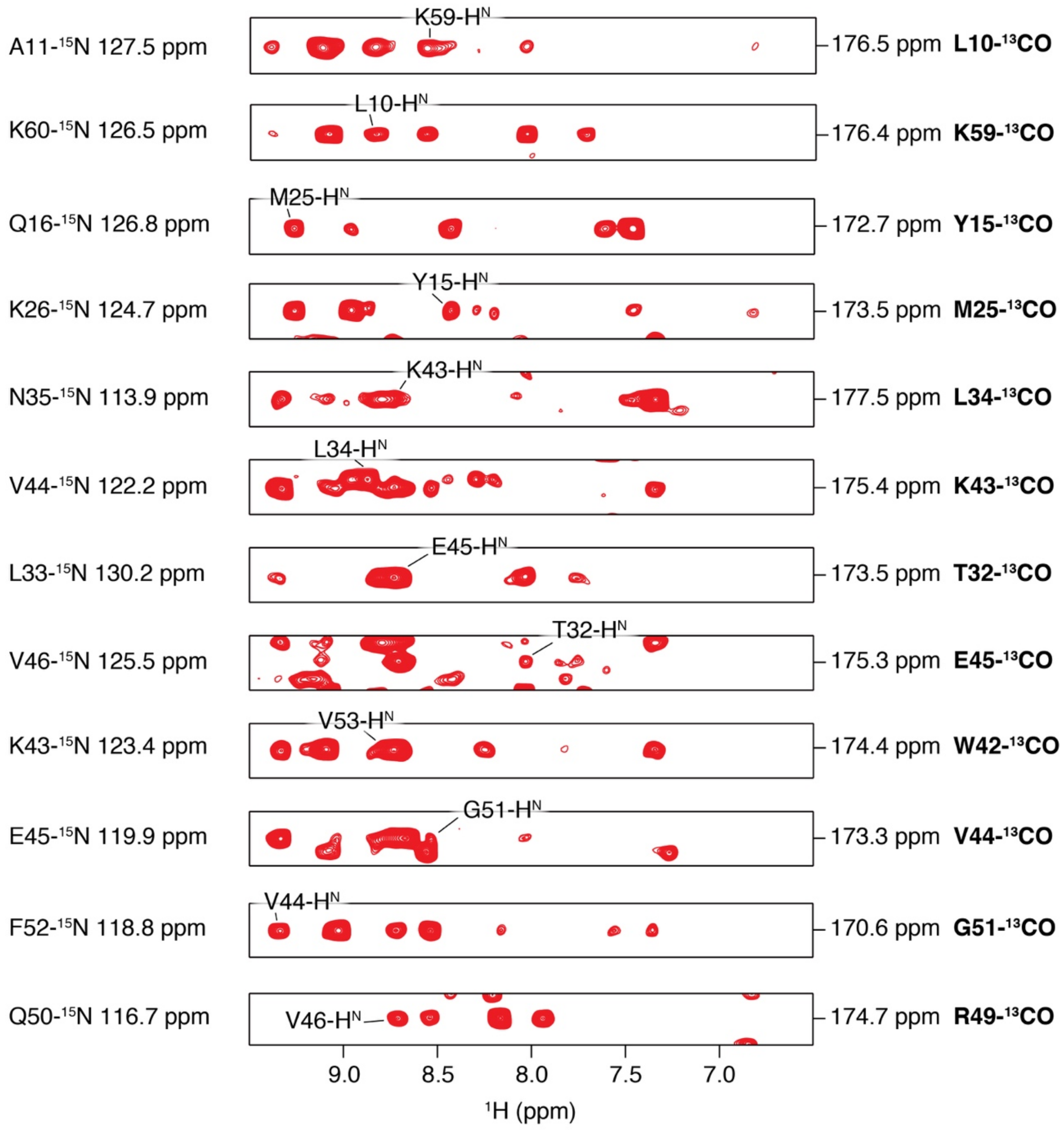

Supplementary Figure 3. Selected 2D planes of the 3D (H)NCOH spectrum of SH3. Cross peaks reflecting hydrogen bonds between amide protons and carbonyls are labeled with the respective amino acid types and their sequence number.

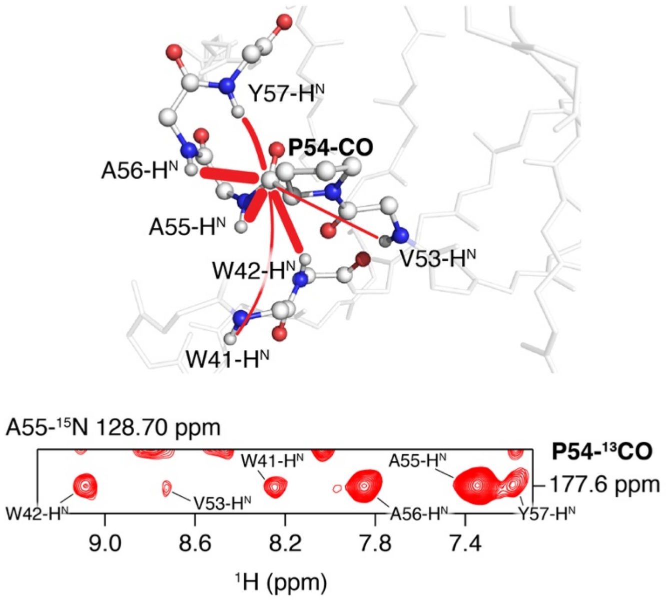

Supplementary Figure 4. Detection of a proline carbonyl in SH3. The H^N of amino acids close in space can be used as probes to observe the CO resonances of prolines. The lines in the structural illustration indicate observed interactions and their thickness reflects the cross peak intensities.

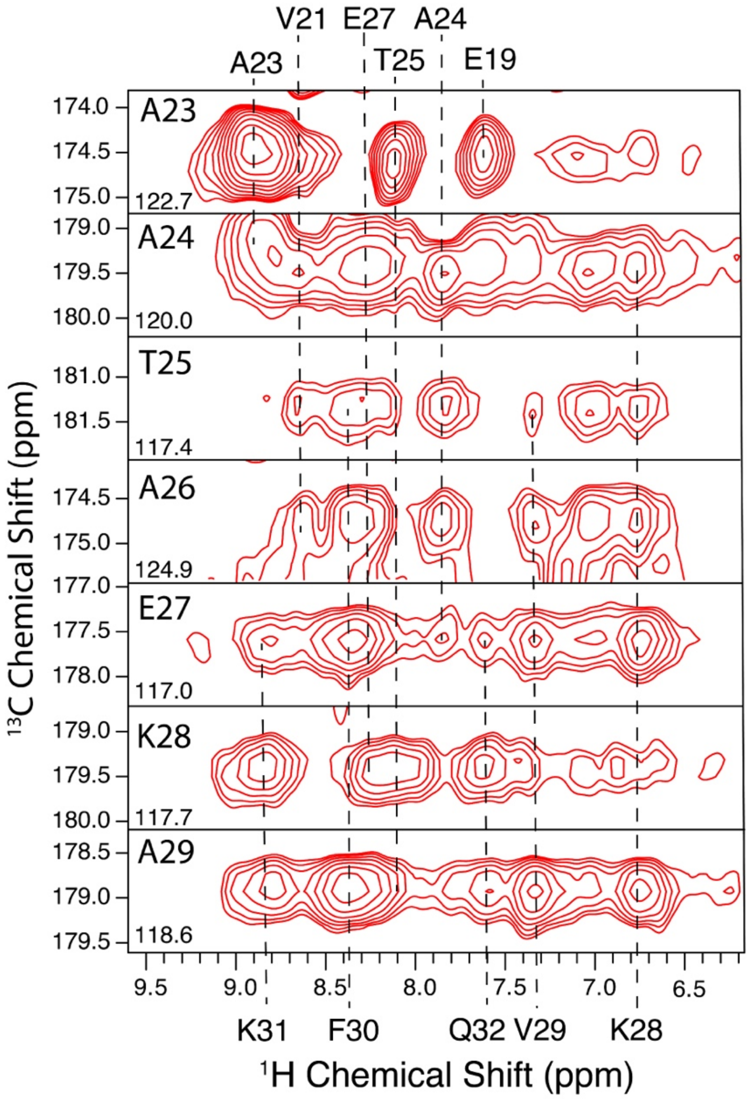

Supplementary Figure 5. Selected 2D planes of the 3D (H)NCOH spectrum of GB1. As examples, cross peaks of residues in the α-helix of GB1 are shown.

Supplementary Table 1. Distances between H^N, carbonyl- and α-carbons in secondary structure elements of proteins. Distances (given in Å) between the indicated atoms are based on idealized secondary structures: α-helix (PDB code 4U1H (Kløverpris et al., 2015)), and parallel and antiparallel β-sheets (PDB codes 2LBU and 2LNQ, respectively (Qiang et al., 2012; Schütz et al., 2011)).

α-helix [Å]		β-sheet (parallel) [Å]		β-sheet (antiparallel) [Å]	
$CO_i \cdots H^N_{i-1}$	5.5				
$CO_i \cdots H^N_i$	3.2	$CO_{i-1} \cdots H^N_j$	3.1	$CO_{i-1} \cdots H^N_i$	2.0
$CO_i \cdots H^N_{i+1}$	2.0	$CO_i \cdots H^N_j$	4.4	$CO_j \cdots H^N_i$	3.2
$CO_i \cdots H^N_{i+2}$	3.2	$CO_{j-1} \cdots H^N_j$	2.0	$CO_i \cdots H^N_i$	2.6
$CO_i \cdots H^N_{i+3}$	3.1	$CO_j \cdots H^N_j$	2.8	$CO_{j-1} \cdots H^N_i$	5.4
$CO_i \cdots H^N_{i+4}$	3.2				
$CO_i \cdots H^N_{i+5}$	4.9				
$C\alpha_i \cdots H^N_{i-1}$	4.8				
$C\alpha_i \cdots H^N_i$	2.0	$C\alpha_{i-1} \cdots H^N_j$	4.4	$C\alpha_{i-1} \cdots H^N_i$	2.6
$C\alpha_i \cdots H^N_{i+1}$	2.6	$C\alpha_i \cdots H^N_j$	3.3	$C\alpha_i \cdots H^N_i$	2.1
$C\alpha_i \cdots H^N_{i+2}$	4.1	$C\alpha_{j-1} \cdots H^N_j$	2.5	$C\alpha_{j+1} \cdots H^N_i$	4.2
$C\alpha_i \cdots H^N_{i+3}$	3.8	$C\alpha_j \cdots H^N_j$	2.1	$C\alpha_j \cdots H^N_i$	4.2
$C\alpha_i \cdots H^N_{i+4}$	4.2				
$C\alpha_i \cdots H^N_{i+5}$	6.3				
